# Response to uncertainty management in regulatory and health technology assessment decision-making on drugs: guidance of the HTAi-DIA Working Group – author’s reply

**DOI:** 10.1017/S0266462323002817

**Published:** 2022-12-18

**Authors:** Milou Amber Hogervorst, Rick Vreman, Inkatuuli Heikkinen, Wija Oortwijn

**Affiliations:** 1 Utrecht University, Division of Pharmacoepidemiology and Clinical Pharmacology, Utrecht Institute for Pharmaceutical Sciences (UIPS), Utrecht, The Netherlands; 2 MSD, Global Regulatory Policy, Copenhagen, Denmark; 3 Radboud University Medical Centre, Department for Health Evidence, Nijmegen, The Netherlands

We welcome the interest of Grimm et al. (1) in the guidance of the HTAi-DIA Working Group that intends to support stakeholder deliberation on the systematic identification and mitigation of uncertainties in the regulatory-HTA interface (2). In their letter, the authors put forth two arguments. First, they state that “it remains unclear if and how the state of the art on uncertainty in HTA was used to develop the guidance”, specifically that their paper “TRUST tool 2020 (3) was not cited”, and that it “bears non-negligible similarity” with the building blocks comprising decision-making uncertainty of the guidance. In essence, this comment relates to the process of developing the guidance. Second, they state that “it is unclear how the presented guidance improves upon this [the TRUST tool 2020].” As authors involved in the process of developing the HTAi-DIA guidance, we would like to reflect on these arguments.

Regarding the process of developing the guidance, we would like to reiterate that the guidance builds on the recommendations of the 2021 HTAi Global Policy Forum (4). It was developed by a cross-sectoral, interdisciplinary Working Group using a deliberate structured process. The steps and various methods deployed are documented in detail in the article (2). We fully agree with the commentators that much has been written about uncertainty in and outside of the HTA domain. The literature that served as a basis for the discussions of the Working Group was, therefore, collected as part of the scoping process to determine the breadth of available information. It was – as clearly mentioned in the article (2) – not a systematic, but a scoping review. The scoping review in combination with iterative, multi-stakeholder dialogues did, nevertheless, result in similar relevant topics that the commentators also mention in their response (i.e., identification, analysis and communication of uncertainty, and risk tolerance). Details on the scoping review and the included papers, among them the clearly cited TRUST tool 2020, are publicly available as part of the guidance (2). Furthermore, 10 of the papers referenced by the commentators as missed are cited in our scoping review (3;5–13), whereas four of the papers mentioned by the commentators were published after completion of the final Working Group meeting – as described in the guidance – in September 2022 (14–17). The remaining papers mentioned by the commentators focus on health economic modelling, managed entry agreements, non-HTA or regulatory papers, which were not in scope for the purpose of the Working Group’s exercise.

Based on the majority of articles that were referenced by the commentators (i.e., focusing on health economic modeling, including the TRUST 2020 tool) and the topics that they considered to be missing (e.g., the scope and specificity of the methods for identifying and analyzing uncertainty), we believe that they – researchers from the Netherlands with a background in health economics – have a distinctly different perspective on approaching and addressing uncertainty in decision-making. The added value of the guidance is clear considering the emphasis on capturing different perspectives, and we consider the need to further stress the well-recognized importance of (early) dialogue between different stakeholders to ensure that there is mutual understanding and common language regarding uncertainty. Furthermore, the HTAi-DIA guidance acknowledges *all* relevant regulatory and HTA uncertainties which can be used in a proactive, rather than a reactive way. This means that besides improving mutual understanding, our guidance could be used to have discussions on the tools used to identify uncertainty, including, for example, the TRUST 2020 tool.

The guidance has been well-received by stakeholders across the HTA ecosystem, with a clear interest in using it in the regulatory-HTA interface. The HTAi-DIA Working Group is, therefore, confident to continue to address relevant topics in this field, such as applying the guidance in combinations of diagnostics and drugs and/or diagnostics and medical devices or medical devices only. Finally, as mentioned during all our communications, the HTAi-DIA Working Group always welcomes input from the broader HTA community and beyond, and we are open to those who wish to become active members of it.
